# Field Performance Assessment of Irradiated *Aedes albopictus* Males Through Mark–Release–Recapture Trials With Multiple Release Points

**DOI:** 10.3389/fbioe.2022.876677

**Published:** 2022-07-19

**Authors:** Fabrizio Balestrino, Arianna Puggioli, Marco Malfacini, Alessandro Albieri, Marco Carrieri, Jeremy Bouyer, Romeo Bellini

**Affiliations:** ^1^ Centro Agricoltura Ambiente “G. Nicoli”, Sanitary Entomology and Zoology Department, Crevalcore, Italy; ^2^ FAO/IAEA Insect Pest Control Laboratory (IPCL), FAO/IAEA Agriculture and Biotechnology Laboratories, FAO/IAEA Joint Division of Nuclear Techniques in Food and Agriculture (NAFA), Vienna, Austria; ^3^ CIRAD, UMR ASTRE CIRAD-INRA, Animal, Health, Territories, Risks and Ecosystems, Montpellier, France

**Keywords:** sterile insect technique (SIT), dispersal, survival rate (S), normalized difference vegetation index (NDVI), sterile to wild male ratio (S/W)

## Abstract

Mark–release–recapture (MRR) trials have been conducted in Northern Italy to evaluate the capacity of radio-substerilized *Aedes albopictus* males to survive, disperse, and engage in mating in the field. Two MRR sessions with the human landing collection method (HLC) were conducted with the simultaneous release of irradiated males marked with four different pigment colors. The survival and dispersal rates seem to be influenced more by environmental factors such as barriers, shading, and vegetation rather than weather parameters. In this study, we confirmed a positive linear relationship between the sterile adult male’s daily survival rate and the relative humidity previously reported in similar experimental conditions and a different dispersal capacity of the released *A. albopictus* males in low- (NDVI index <0.4) and high (NDVI index >0.4)-vegetated areas. Consistent with previous studies, *A. albopictus* males have their maximal dispersion in the first days after release, while in the following days the males become more stationary. The similar field performances obtained with marked and unmarked radio-sterilized and untreated *A. albopictus* males on similar environments confirm the negligible effects of irradiation and marking procedures on the quality of the males released. The similar sterile to wild (S/W) male ratio measured in high- and low-vegetation areas in the release sites indicates a similar distribution pattern for the wild and the released sterile males. According to the MRR data collected, the Lincoln index estimated different *A. albopictus* mean population densities in the study areas equal to 7,000 and 3,000 male/ha, respectively.

## Introduction

The control of urban mosquitoes such as *Aedes aegypti* and *Aedes albopictus* is still an unresolved worldwide problem, as clearly demonstrated by the official number of cases due to the diseases they transmit, such as dengue, Zika, chikungunya, and yellow fever (WHO 2017). The currently applied mosquito control strategies are not achieving the expected results, and the rise of resistance detected in many regions against several key insecticides is alarming and shadowing on the future capacity to fight mosquito vectors (Ranson et al., 2010; Vontas et al., 2012; Grigoraki et al., 2015). Therefore, new effective tools or strategies to be integrated into the existing ones are under development, including genetic manipulation of vector species, *Wolbachia*-based technologies, autodissemination, and the sterile insect technique (SIT) (Flores and O’Neill 2018; Achee et al., 2019).

The SIT is a species-specific and environment-friendly method of pest control using sequential inundative releases of radio-sterilized males to reduce the reproduction capacity of the target population (FAO 2005). The use of radiation to produce dominant lethal mutations to generate sterile or sub-sterile adult insects has a long history of success (CJEU, 2018; Dyck et al., 2021), has a remarkable lack of resistance (Alphey et al., 2010; Bull 2015), and does not involve the release of insects modified through transgenic engineering processes.

In the past years, a considerable development of SIT application against mosquitoes has been implemented against *A. albopictus* in Italy, and pilot field trials have been conducted in several urban localities to test the performances of sterile males in real condition (Bellini et al., 2013a; Bellini et al., 2021).

In order to integrate the SIT into operational area-wide mosquito management programs, it is essential to verify if mass production, sterilization, and release procedures adopted can negatively affect the quality of the males imposing critical costs. Irradiated sterile males were found to be equivalent to fertile wild competitor under laboratory and semi-field conditions (Bellini et al., 2013b; Madakacherry et al., 2014; Damiens et al., 2016), while the field estimation of the competitiveness index of radio-sterilized *A. albopictus* males under field conditions resulted in a range of 0.03–0.38 (Bellini et al., 2021). One of the most used parameters to measure the effect of the sterile male releases on population suppression is the mating competitiveness value (Fried, 1971) which directly depend on the sterile to male ratio obtained in the field (Bellini et al., 2021). The field evaluation of the sterile to wild male ratio (S/W) provides important indications to modulate the dose and frequency of releases to avoid prolonged period of insufficient presence of sterile males in the field (Hendrichs et al., 2005). The present study was conducted as a collaboration between CAA and the Department of Technical Cooperation of the International Atomic Energy Agency (TC-IAEA) to examine the effectiveness of *A. albopictus* radio-sterilized males to disperse, survive, and effectively compete for mating. The activities described report on the analysis of the survival and dispersal capacities of males during an integrated Aedes vector control program with an SIT component using mark-release-recapture trials with multiple release points.

## Materials and Methods

*Mosquito rearing and sterilization procedures*. The mosquito strain used in these trials was started from field materials collected in urban areas of Rimini, Emilia-Romagna (Italy) and maintained for many generations (RN strain F68–F72), under standard laboratory conditions (28 ± 1°C, 80 ± 5% RH, L:D 14:10 h) within the mass rearing pilot module of the Sanitary Entomology and Zoology EZS Department, CAA “G. Nicoli” (Crevalcore, Italy). The rearing methods and conditions used were the same as those described in Balestrino et al. (2017). *Aedes albopictus* male pupae were sorted using metal sieves with square holes of 1,400 μm size, at 24–30 h after the beginning of pupation (Medici et al., 2011), and aged for additional 24 h before irradiation treatment (pupal age at irradiation 24–48 h). Irradiation was performed at the Medical Physics Department of the St. Anna Hospital (Cona, Ferrara, Italy), with a dose of 35 Gy using an IBL 437 irradiator (CIS Bio International, France) equipped with a Cs-137 linear source with a central dose rate of 1.70 ± 3.5% Gy/min (Balestrino et al., 2010). The routine dosimetry and the dose distribution inside the basket are routinely checked using GAFCHROMIC EBT3 dosimetry films (International Specialty Products, Wayne, NJ). After irradiation, the males were transferred back to the laboratory and then placed for emergence inside dedicated cardboard boxes (12 × 12 × 18 cm) with continuous access to cotton pads soaked in a 10% sucrose solution. The radiation dose of 35 Gy was selected as the most effective radiation dose capable to induce in *A. albopictus* a residual fertility of about 1% while maintaining effective flight capacity and quality parameters (Balestrino et al., 2017). In *A. albopictus*, the residual fertility observed at 35 Gy do not affect the mating competitiveness and do not reduce the effectiveness of the technique (Bellini et al., 2013a; Bellini et al., 2021).

**FIGURE 1:**
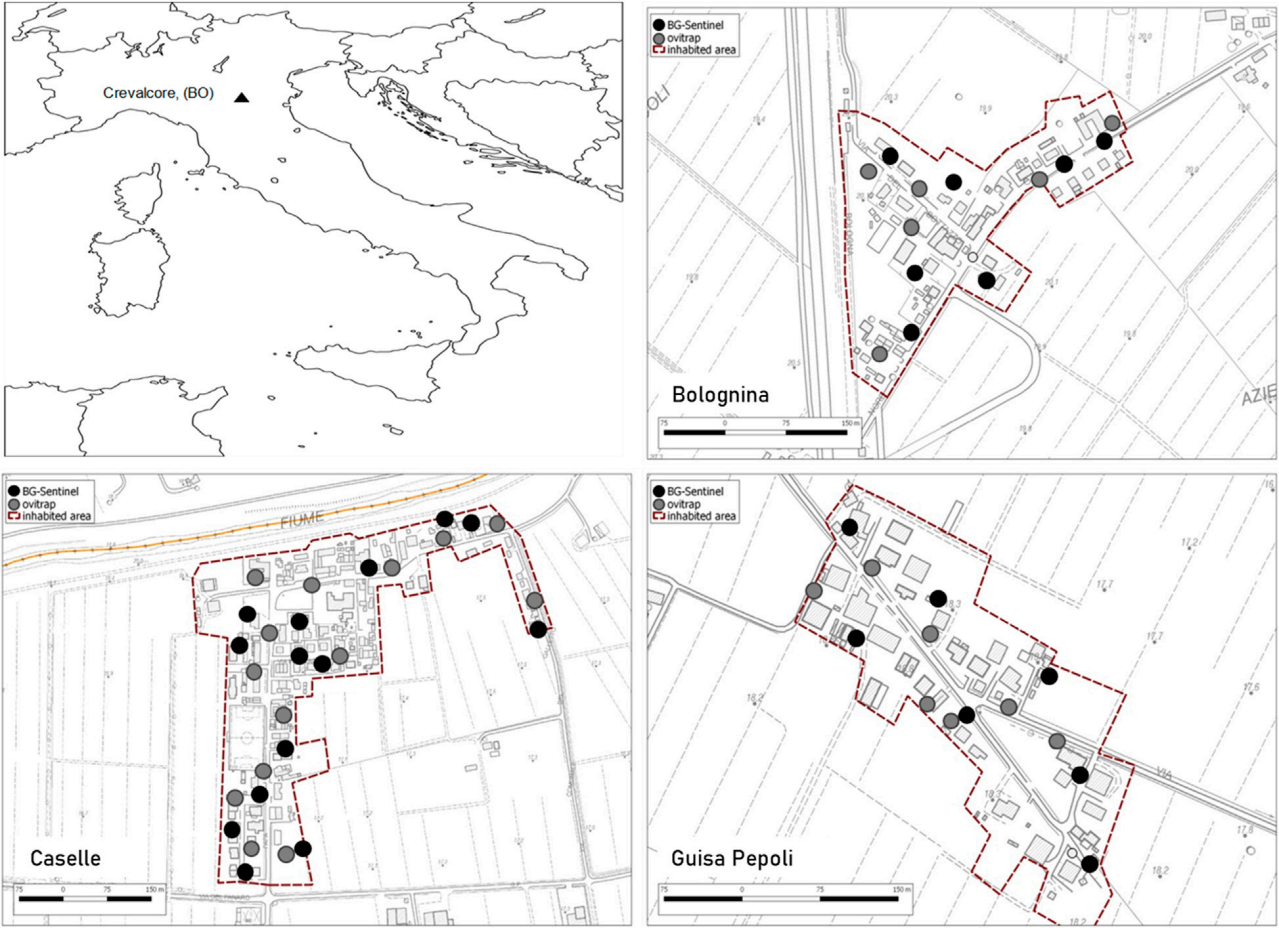
Study areas in Crevalcore, Bologna, Emilia-Romagna, Italy, with indication of the georeferenced monitoring stations (BG-sentinel and ovitraps) in each study localities.

*Study area.* The field trials were conducted in three suburban localities close to the CAA facility: Caselle (44.789718 N, 11.171939 E), Guisa Pepoli (44.702212 N, 11.167757 E), and Bolognina (44.763999 N, 11.146243 E) situated in the municipality of Crevalcore, Bologna province, Northern Italy (Figure 1). The villages selected have similar median human population density of about 30 inhabitants per ha and size of 14, 7, and 7 ha, respectively. Each locality is surrounded by rural areas and included usually two-storied houses, separated by narrow lanes, with many private and some public gardens. Larval treatment of permanent breeding sites and removal or inactivation of occasional breeding sites were conducted on monthly basis from May to September 2019 in the three villages in public and private areas (door-to-door campaign) without the application of adulticides treatments (Canali et al., 2017; Donati et al., 2020).

*Weather and environmental parameters.* Weather parameters such as air temperature, relative humidity, wind (speed and direction), and rainfall were recorded throughout the course of the study by a weather station situated in Sant’ Agata Bolognese (Dext3r data; Regional Agency for Environmental Protection Emilia-Romagna Region, ARPAE) a few kilometers from the three study localities. The Normalized Difference Vegetation Index (NDVI) has been used to characterize the study areas for vegetation cover. The NDVI is an indicator of the greenness of the biomes, and two NDVI threshold values able to identify the vegetation types in an urban area were defined; from 0.4 to 1 indicates the medium-high vegetation like trees in urban areas, while a value lower than 0.4 represents low vegetation or non-vegetation. NDVI was calculated from Landsat eight images (https://ers.cr.usgs.gov/) with 30 m resolution, and QGIS 3.16 was used to extract data values on HLC georeferenced points.

*Monitoring system.* In all localities**,** a monitoring system at a density of two monitoring stations per hectare was implemented a week before the first male release. Each monitoring station was composed by one BG-sentinel trap baited with standard BG-lure (BG-sentinel 2, Biogents, Regensburg, Germany) and one ovitrap (CAA14GR; Carrieri et al., 2017). Ovitrap consists of 1.4 L black plastic container holding 800 ml of dechlorinated water and a strip of masonite (15 × 2.5 cm) as egg deposition substrate. BG-sentinel traps were activated in the early afternoon at day 7 from the release and operated for 24 h, while ovitraps were placed on the release day, operated continuously, and checked at day 7 from release (Figure 1). The mosquito population dynamics was monitored in the release and control areas by counting and hatching the eggs collected in the ovitraps monitoring system. The eggs were counted under a stereomicroscope, embryonated for 7 days, and hatched using a standard procedure to assess their fertility rate in the release areas in comparison with the control area (Bellini et al., 2013a).

**FIGURE 2:**
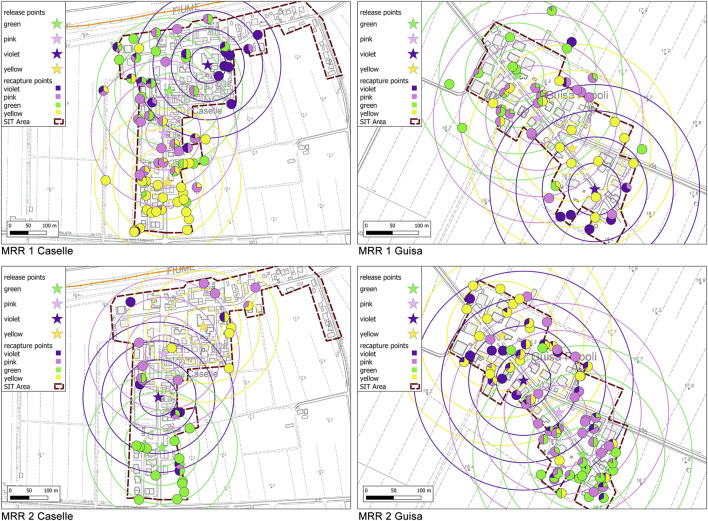
Maps of the recapture points (HLC–circles) located into concentric annuli of 50 m up to the maximum of 200 m radius around the release stations (stars) defined during the different MRR trials in Caselle and Guisa Pepoli. The HLC circles are colored according to the percentage of recapture of males of different colors collected in each station.

*MRR trials.* To estimate the survival and the dispersal of released sterile males and to measure the sterile to wild male ratio (S/W), two MRR studies were undertaken on July 06–13 and August 03–10, 2019 in the localities of Caselle and Guisa Pepoli. Sterile marked males were released in the MRR areas only (Caselle and Guisa Pepoli), while the recapture sessions were performed in the release and in the control localities (Bolognina). The sterile adult males released in the MRR areas (age 36–48 h) were equally divided and marked while held in the release cardboard boxes using a manual insufflator just before the release, using fluorescent powders of four different colors (Zuper Paint Fluorescent; pink, violet, green, and yellow dust) at a dose of 0.3 g per 1,000 adult (FAO/IAEA 2020). Marked sterile males were ground released by subsequentially opening the cardboard boxes at the four selected release stations employing a different color for each station (Table1; Figure 2). Distance between release stations was in the range 80–130 m. The release stations selected were usually exposed to the Sun and the releases were performed in the late morning. Mortality of male adults was checked by counting the dead and the not escaped adults observed in the cardboard boxes after 30 min from opening. For each release, a sample of about 300 pupae was withdrawn to check the consistency of the residual presence of females. In each locality, 24 sampling stations distributed to cover an area of about 200 m radius from each release point were randomly selected in each locality and assigned daily to two operators. In total, 12 operators were rotated daily to avoid potential collection bias. The human landing collection (HLC) sessions were conducted daily starting from the first day after release for seven consecutive days, from 5:00 p.m. to 7:00 p.m. Adult males and females of *A. albopictus* approaching the operator were collected using a manual battery-operated aspirator for 5 min in each sampling station. Each operator was provided with a large black plastic bag filled with polystyrene pieces (30 × 40 × 50 cm; LxWxH) to standardize its visual attraction toward mosquito males and was also provided with a container to transport the adult samples collected. The adult mosquitoes collected were stored overnight at −20°C and screened for identification of species, sex, and marking color the following day under a stereomicroscope using an UV light source. The coordinates of release and recapture stations were entered into an open-source Geographical Information System (QGIS 3.16), to calculate the distance and direction (angles) between each release and recapture site and create thematic maps.

**TABLE 1:** Main descriptive data of the MRR trials from rearing to release (Release). NP, number of pupae processed; SA, separation accuracy (sexing method); NP_M_, number of pupae male; ER, emergence rate; NA_M_, number of adult male; MR, mortality rate of the marked male at release; NA_M__R, number of adult male released; NA_M__R/ha, number of adult male released per hectar (ha) used in the two localities of Caselle (14 ha area) and Guisa Pepoli (7 ha area) during the first and second MRR trial. In HLC is reported the overall number of wild (W_M_ male and W_F_ female) and sterile marked adults (Y_M_, yellow male; V_M_, violet male; G_M_, green male; and P_M_, pink male) recaptured during the human landing recapture sessions in the different MRR trials. Sterile males were released in Caselle and Guisa Pepoli, while the recapture sessions were performed in all localities. The sterile to wild ratio (S/W) and the sex ratio (SR) calculated in the experimental areas are also reported. In ovitrap, the mean (M ± SD) number of eggs (NE), hatching rate (HR), and the relative Fried male competitiveness index (F_INDEX_) calculated for each release localities are reported (See also Supplementary Materials S1–S4).

Parameter	Date	Area	MRR	NP	SA	NP_M_	ER	NA_M_	MR	NA_M__R	NA_M__R/ha
Release	06/07/18	Caselle	1	15,000	0.986	14,811	0.910	13,485	0.053	12,768	912
	06/07/18	Guisa	1	7,000	0.986	6,916	0.925	6,397	0.050	6,079	869
	03/08/18	Caselle	2	7,000	0.981	6,867	0.985	6,766	0.052	6,417	458
	03/08/18	Guisa	2	19,000	0.981	18,639	0.976	18,192	0.043	17,404	2,486
**Parameter**	**Date**	**Area**	**MRR**	**W_M_**	**Y_M_**	**V_M_**	**G_M_**	**P_M_**	**W_F_**	**S/W**	**SR**
HLC	07_13/07/18	Caselle	1	756	98	49	42	59	490	0.33	1.54
	07_13/07/18	Guisa	1	357	32	9	83	71	351	0.55	1.02
	07_13/07/18	Bolognina	1	740	-	-	-	-	798	-	0.93
	04_10/08/18	Caselle	2	1,013	9	12	26	13	542	0.06	1.87
	04_10/08/18	Guisa	2	441	116	72	86	121	271	0.90	1.63
	04_10/08/18	Bolognina	2	1,028	—	—	—	—	783	—	1.31

**Parameter**	**Date**	**Area**	**MRR**	**N**	**NE (M ± SD)**		**HR (M ± SD)**			**F _INDEX_**	
Ovitrap	13/07/18	Caselle	1	14	264.6	±157.8	0.834	±0.103	**NS**	0.25
	13/07/18	Guisa	1	7	206.9	±139.0	0.791	±0.166	**NS**	0.26	
	13/07/18	Bolognina	1	7	348.6	±178.8	0.904	±0.044		
	10/08/18	Caselle	2	14	409.9	±179.9	0.902	±0.045	**NS**	0.49
	10/08/18	Guisa	2	7	179.3	±112.7	0.671	±0.135	***	0.43
	10/08/18	Bolognina	2	7	534.7	±225.7	0.928	±0.028		

## Statistical Analysis

*Monitoring system.* Linear regression analyses were run to evaluate the relationship between the weekly number of eggs collected in ovitraps and the adults collected with HLC during MRR trials as well as between the weekly number of eggs collected in ovitraps and the adult collected in BG-sentinel traps in the study areas. The relationship between the presence of sterile and wild adult mosquito collected in HLC and the vegetation cover (NDVI Index) was investigated in Guisa Pepoli and Caselle during the second MRR trials. QGIS was used to create an inverse distance weighted (IDW) interpolation raster for marked males in the second MRR session in Guisa Pepoli where we collected a data set sufficient to support statistical evidence. IDW interpolation method was applied to predict the MDT and the S/W values over the entire study area using the measured values surrounding the prediction locations. The measurement of the sterile to wild males’ ratio (S/W) was estimated by assessing the mean ratio between marked (sterile) and unmarked (wild) captured males performed the human landing collections (HLC). IDW assumes that each measured point has a local influence on the prediction location that diminishes with distance.

*MRR trials.* The sterile male dispersal pattern was summarized by the mean distance traveled (MDT), maximum distance traveled (MAX), and flight range (FR) for each locality. Dispersal distance of *A. albopictus* males (MDT) was measured by drawing annuli 50 m apart around the release sites and applying a correction factor to account for unequal trap densities (MDT) as previously described (Le Goff et al., 2019; Supplementary Materials S1–S4). Linear regression analyses were performed to evaluate the relationship between the daily distance traveled (MDT) by the sterile males and the day after the release. ANOVAs were performed to evaluate differences in MDT among the two study areas and the NDVI index. The FR was estimated through the linear regression of the cumulative estimated recaptures performed within each annulus (*x*-axis) on the log10 (annulus median distance +1). The FR_50_ and FR_90_ indicate the distance that comprehends the maximum flight distance reached by 50% and 90% of the individuals, respectively. These parameters were calculated from the equation of regression as the value of y at 50% and 90% of the largest value of x, respectively. The mean angle of dispersion (MAD) from the release point (a) was calculated for each locality of release. For each mean angle, the length of the mean vector (r) and the angular deviation (s) was calculated to determine the presence of preferential dispersal directions (from 0 non-directional dispersion to 1 unidirectional dispersion). To determine whether the dispersal direction differed significantly from non-directional uniformity, Rayleigh’s test was applied (Bellini et al., 2010; Supplementary Materials S1–S4). The probability of daily survival (PDS) was estimated by regressing log10 (x +1) of the number of recaptures against the day of recapture where the antilog_10_ of the slope of the regression line is the PDS. Average life expectancy (ALE) was calculated from the PDS as 1/-loge PDS. The linear corrected method was used to estimate the survival rate. The recapture rates *Θ* and survival S rates were estimated with equations *Θ* = *e*^a/(N + *e*^a) and S = *e*^b/(1-*Θ*)^1/d, respectively, where a and b were the regression coefficients of the linear regression of the ln-transformed captures as a function of time; N is the number of individuals released; *Θ* is the recapture rate; day is the number of days after release; and S is the daily survival rate (Bellini et al., 2010; Supplementary Materials S1–S4).

The model that estimates the sterile male survival rate as a function of the daily relative humidity (RH) previously observed with fertile males in similar environment and described by the formula S = 0.021 RH−0.48 (Bellini et al., 2010; Bellini et al., 2021) was compared with the data observed in the MRR field trials using a *t*-test analysis. The one-way ANOVA of the daily survival rate and mean distance traveled data were compared with MRR data obtained in 2010 with *A. albopictus* fertile males marked with fluorescent pigments or by eliminating *Wolbachia* symbiont (aposymbiotic strain) in suburban localities with similar environmental conditions (Bellini et al., 2010).

The Lincoln index modified for a low recapture rate and compensated for daily survival (P) was used to estimate the wild male population size for the different batches used in each locality at each day after release. The modified Lincoln index is calculated as P = [R*St (n-m+1)]/(m+1), where S is daily survival rate and t is sampling day after release, R is the number of marked males, n is the total number of recaptures of both marked and wild adult males, and m is the number of recaptured marked males (Le Goff et al., 2019; Supplementary Materials S1–S4).

The fertility rate of the eggs collected in the release and control sites allow to estimate the sterile male competitiveness index under field conditions using the Fried competitiveness index (Fried, 1971) calculated as F = W/S * [(PW–PS)/(PS–PRS)], where PW is the mean natural fertility in the control site of Bolognina, PS is the fertility rate observed in the release area, and PRS is the residual fertility of the released males. The PRS was close to zero and always below 1% and therefore we not considered this correction in the competitiveness index formula (Bellini et al., 2013a; FAO/IAEA 2020, Supplementary Materials S1–S4). All statistical analyses were conducted using STATISTICA 7.0 software package (StatSoft Inc., United States) and are reported in the Supplementary Materials S1–S4.

## Results

*Monitoring system*. The weekly mean number of eggs per ovitrap (E) shows a significant linear relationship with the number of wild males (M_HLC_ = 0.011 *E + 0.265; *R* ^2^ = 0.83; F_1,4_ = 19.1; *p* = 0.012) and wild females (F_HLC_ = 0.008 *E + 0.62; *R* ^2^ = 0.69, F_1,4_ = 9.0; *p* = 0.039) observed in the study area by the HLC collections while no correlation was observed between HLC adult collection and BG-sentinel traps catches, either for wild females (*R* ^2^ = 0.69; *p* = 0.13), wild males (*R* ^2^ = 0.14; *p* = 0.79), or released sterile males (*R* ^2^ = 0.35; *p* = 0.65).

*MRR trials.* In the localities of Caselle and Guisa Pepoli, about 19,000 and 23,500 marked males were released in the two MRR trials (Table 1), respectively, with a residual presence of females equal to 1.65% (±0.36%) on the total number of released adults. The mean (±SD) mortality rate observed following the release of sterile marked males was equal to 4.9 (±0.4%) (Table 1), which was similar to the mortality rates reported in the literature during MRR trial with fertile *A. albopictus* males marked with fluorescent dust (Bellini et al., 2010; Gouagna et al., 2015). The overall released males in the different MRR trials and the HLC mean recapture data are summarized in Table 1 and further data are reported in the Supplementary Materials S1–S4. The maps of the release and recapture points in the two MRR trials for each locality are shown in Figure 2.

**FIGURE 3:**
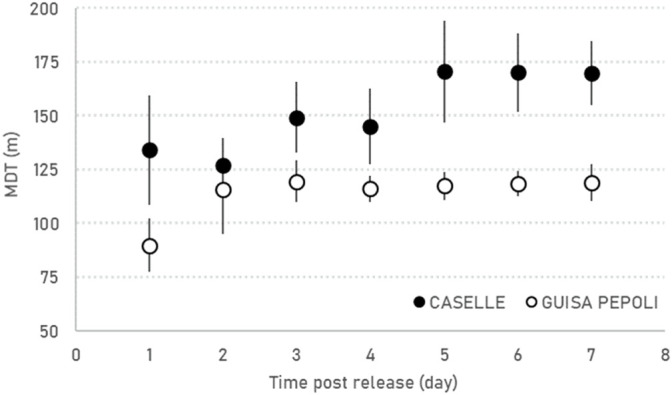
Daily mean (±SE) distance traveled (MDT) by sterile marked males recorded in the MRR trials in Caselle and Guisa Pepoli.

The sterile males have similar MDT increase rate over time in the two localities but in Caselle the males showed a significant (F_1,14_ = 4.64; *p* = 0.049) higher MDT (172.9 ± 45.8 m) in comparison with Guisa Pepoli (MDT 126.4 ± 44.6 m) (Table 2; Figure 3). The sterile males showed their maximal daily dispersion on day one post-release, while in the following days, the sterile males continue to disperse from the release station with a lower and constant intensity (Table 2; Figure 3). The mean (±SD) distance traveled (MDT) by the sterile males at the third day after release in Guisa Pepoli was about 95.9% (±2.8%) of their mean dispersal capacity (MDT), while in Caselle the sterile males covered 97.4% (±3.7%) of their overall displacement at the fifth day after release. The overall mean (±SD) distances within which the 50% (FR_50_) and 90% (FR_90_) of the released males were re-collected and the maximal distance (MAX) traveled by males in Caselle (FR_50_ = 115.3 ± 42.2 m; FR_90_ = 283.7 ± 112.5 m; MAX = 304.5 ± 113.1 m) are all higher than values measured in Guisa Pepoli (FR_50_ = 76.0 ± 35.7 m; FR_90_ = 227.0 ± 74.8 m; MAX = 249.8 ± 45.2 m) but not significantly different (FR_50_: *p* = 0.064; FR_90_: *p* = 0.255; MAX: *p* = 0.224).

**TABLE 2:** Dispersion and survival parameters registered in the two SIT localities of Caselle and Guisa Pepoli in the first and second MRR trials. *MDT*, mean distance traveled; *MAX*, maximum distance traveled; *FR50%* and *FR90%*, maximum flight distance reached by 50% and 90% of the individuals; *MAD*, mean angle of dispersion (r, length of dispersion; s, angular deviation; a, estimated mean angle; Z value, Rayleigh’s test); *Lincoln index*-*corrected P* indicates the population size expressed in number of male/ha; *PDS*, probability of daily survival; *ALE*, average life expectancy (day); *Θ*, the recapture rate; *S*, the survival rate. *p < 0.05, **p < 0.001, ***p < 0.0001.

Parameter	Locality	MRR	Y_M_	V_M_	G_M_	P_M_	ALL_M_ (M ± SD)
MAX	Caselle	1	395.4	497.1	306.7	336.3	383.9±84.0
m		2	131.7	276.3	193.0	299.7	225.2±77.3
	Guisa	1	174.0	284.8	276.2	231.7	241.7±50.8
		2	320.7	259.3	231.9	219.8	257.9±45.0
MDT	Caselle	1	146.2	189.7	187.0	245.2	192.0±40.7
m		2	111.0	156.9	127.6	219.2	153.7±47.6
	Guisa	1	107.0	188.1	126.4	87.0	127.1±43.7
		2	160.7	172.4	95.3	74.5	125.7±48.1
FR50%	Caselle	1	74.2	126.8	126.4	188.2	128.9±46.6
m		2	68.5	100.2	81.9	156.4	101.8±38.7
	Guisa	1	64.0	128.6	77.5	39.6	77.4±37.5
		2	101.5	113.9	50.6	32.2	74.6±39.3
FR90%	Caselle	1	274.0	313.7	277.9	493.1	339.7±103.8
m		2	121.4	276.6	167.6	345.2	227.7±101.8
	Guisa	1	173.5	312.0	213.5	168.0	216.8±66.7
		2	328.4	300.7	178.3	141.5	237.2±91.2
MAD	Caselle	1	0.4	0.4	0.2	0.1	0.3±0.1
Length of mean vector (r)		2	0.7	0.2	0.3	0.3	0.4±0.2
	Guisa	1	0.4	0.2	0.1	0.6	0.3±0.2
		2	0.2	0.1	0.2	0.3	0.2±0.1
MAD	Caselle	1	64.5	65.2	70.5	76.6	69.2±5.6
Angular deviation (s)		2	48.1	70.6	67.9	69.7	64.1±10.7
	Guisa	1	64.8	70.5	75.4	49.9	65.2±11.1
		2	74.5	75.2	71.4	66.6	71.9±3.9
MAD	Caselle	1	192.6	82.3	258.4	67.0	150.1±91.4
Estimated mean angle (a)		2	111.2	117.8	137.1	192.5	139.6±36.9
	Guisa	1	317.0	171.3	237.0	352.3	269.4±81.3
		2	111.5	52.9	195.9	205.2	141.4±72.5
MAD	Caselle	1	13.15 ***	6.11 **	2.49	0.65	
Z value (Rayleigh’s test)		2	3.78 *	0.7	2.32	0.88	
	Guisa	1	4.16 *	0.53	1.48	27.33 ***	
		2	2.81	1.4	4.30 *	13.82 ***	
Lincoln index corr	Caselle	1	52,048	60,329	117,871	88,038	79,572±29,812
Population size		2	228,119	143,754	45,156	172,380	147,352±76,609
	Guisa	1	12,986	70,514	5,801	7,195	24,124±31,083
		2	11,950	10,669	32,642	17,627	18,222±10,078
Lincoln index cor	Caselle	1	3,253	3,771	7,367	5,502	4,973±1863
Male/ha		2	14,257	8,985	2,822	10,774	9,210±4,788
	Guisa	1	1855	10,073	829	1,028	3,446±4,440
		2	1707	1,524	4,663	2,518	2,603±1,440
PDS	Caselle	1	NA	0.748	0.857	0.777	0.794±0.057
		2	0.871	0.779	0.673	0.825	0.787±0.085
	Guisa	1	0.695	0.837	0.553	0.667	0.688±0.117
		2	0.628	0.600	0.892	0.713	0.708±0.131
ALE	Caselle	1	NA	3,440	6,485	3,971	4,632±1,627
Day		2	7,213	4,004	2,522	5,204	4,736±1,983
	Guisa	1	2,744	5,634	1,690	2,472	3,135±1,725
		2	2,150	1,960	8,747	2,954	3,953±3,225
Recapture rate, Θ	Caselle	1	0.009	0.008	0.004	0.005	0.006±0.002
Per 1 ha released		2	0.003	0.004	0.011	0.004	0.005±0.004
	Guisa	1	0.015	0.002	0.026	0.036	0.020±0.015
		2	0.025	0.033	0.011	0.017	0.022±0.010
Survival rate, S	Caselle	1	0.826	0.733	0.845	0.831	0.809±0.051
		2	0.873	0.783	0.680	0.828	0.791±0.083
	Guisa	1	0.674	0.874	0.568	0.633	0.687±0.132
		2	0.561	0.507	0.731	0.681	0.620±0.104

**FIGURE 4:**
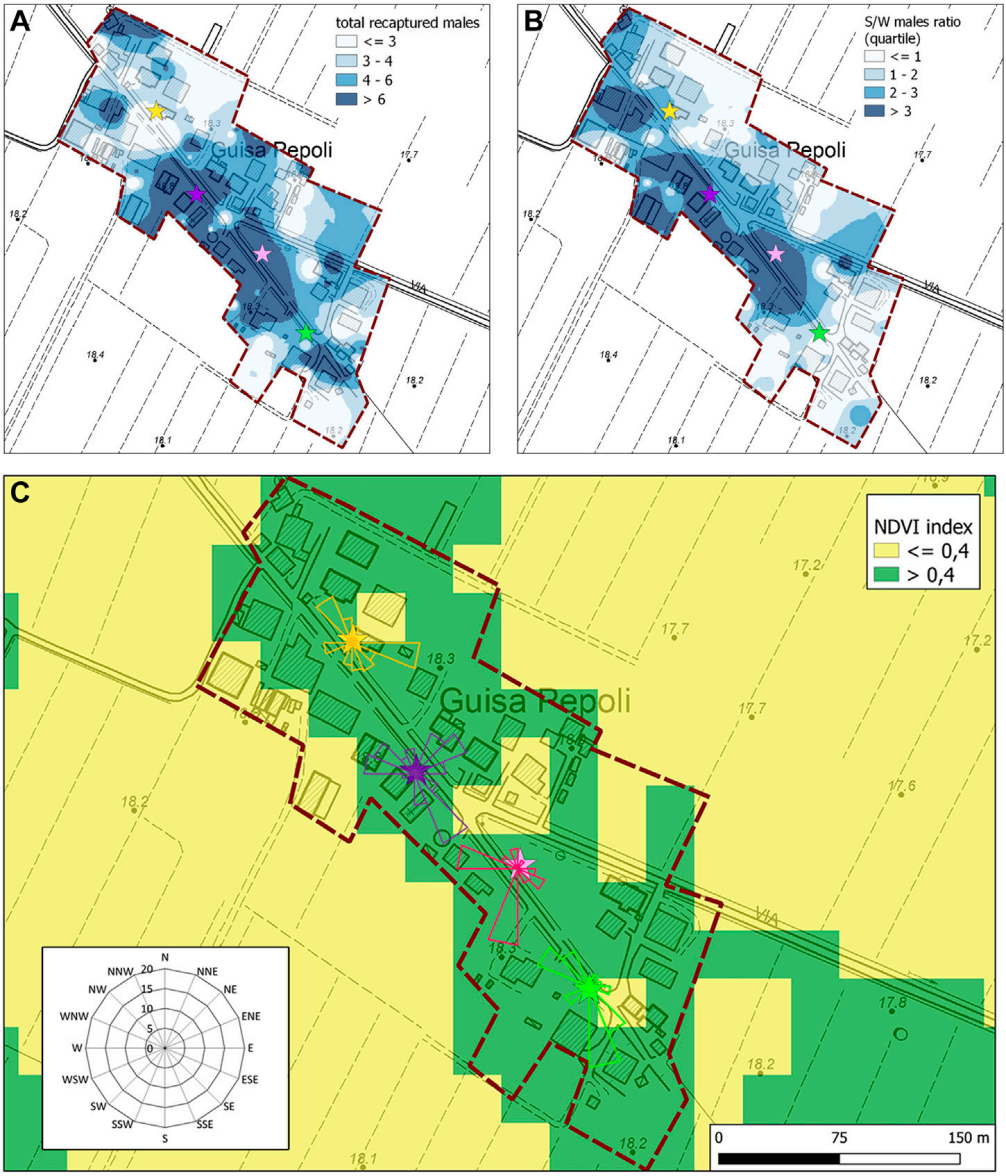
Summary map of **(A)** the total recaptured males and **(B)** S/W ratio of the recaptured males (IDW interpolation rasters) in Guisa Pepoli during the second MRR trial. Colored stars represent the release points. **(C)** Normalized difference vegetation index (NDVI) map and direction dispersion of sterile males in Guisa Pepoli in the second MRR trial. The direction and the dimension of the bands in the rose diagram show the direction and intensity of the sterile male’s distribution.

We also found some evidence of statistically significant preferential dispersion directions with unidirectional tendency in either the first and second MRR trials in both localities without a specific correlation with any weather or environmental parameters (MAD z values in Table 2 and Figure 4A).

The mean NDVI index in the different HLC recollection sites differs significantly (F_1,286_ = 23.8; *p* < 0.0001) among the two MRR areas (Caselle: 0.59 ± 0.15; Guisa Pepoli: 0.51 ± 0.13). The analysis conducted on the second MRR’s data indicate a more uniform distribution of wild and sterile mosquito captures in Caselle with no difference in catches performed in medium–high (>0.4) and low (<0.4) NDVI index for wild female (F_1,166_ = 1.81; *p* = 0.18), wild males (F_1,166_ = 0.44; *p* = 0.51), and sterile males (F_1,166_ = 0.00; *p* = 0.98), respectively. In Guisa Pepoli, the females were uniformly distributed irrespective of the vegetation (F_1,166_ = 0.24; *p* = 0.63), while the number of wild (F_1,166_ = 7.62; *p* = 0.01) and sterile males catches (F_1,166_ = 4.55; *p* = 0.02) were significantly higher in areas with medium–high vegetation index. The sterile male distribution pattern in Guisa Pepoli during the second MRR trial showed a high concentration of males in the central area which is characterized by high NDVI values (Figures 4A, B). While the overall mean sterile to wild male ratio (S/W ± SD) in Guisa Pepoli was equal to 1.24 (±2.83), in the central part of the study area the S/W ratio exceed 3. The similar S/W male ratio measured in Caselle (F_1,125_ = 0.52; *p* = 0.47) and in Guisa Pepoli (F_1,103_ = 0.04; *p* = 0.84) in areas with different vegetation index, indicates a similar distribution pattern for the wild and the released sterile males (Figure 4C).

Results of daily survival probability (PDS) and average life expectancy (ALE) of marked sterile males are presented in Table 2. Whatever the color the mean (±SD) values of the ALE were not different (F_1,13_ = 1.09; *p* = 0.315) in the two localities and varied from 3.54 (±2.43) to 4.69 (±1.69) days in Guisa Pepoli and in Caselle, respectively. The PDS were also similar in the localities tested (F_1,13_ = 3.37; *p* = 0.09) varying from 0.55 to 0.89 with a mean (±SD) value of 0.70 (±0.12) and 0.79 (±0.07), respectively, for Guisa Pepoli and Caselle. The overall mean (±SD) survival rate measured during the MRR trials in Caselle (80.0 ± 6.4%) was significantly higher (F_1,13_ = 9.80; *p* = 0.007) than values observed in Guisa Pepoli (65.4 ± 11.5%). The daily survival rate ranged from 68 to 87% and from 51 to 87% in Caselle and Guisa Pepoli, respectively. The overall mean recapture rate achieved in Guisa Pepoli (2.1 ± 1.1%) was significantly higher (F_1,14_ = 12.5; *p* = 0.003) than the recapture rate obtained in Caselle (0.59 ± 0.3%). The overall cumulative estimation of the mean recapture rates during the MRR trials ranged from 0.26 to 1.08% and from 0.21 to 3.58% in Caselle and Guisa Pepoli, respectively (Table 2).

No differences (*t*-test; *p* = 0.65) were observed between the daily survival rate values (s) directly observed in the MRR trials (0.76 ± 0.06) and estimated based on the RH values (0.74 ± 0.05) using the model described previously. The sterile *A. albopictus* males have an overall higher (F_2,18_ = 7.49; *p* = 0.005) mean daily survival rate in comparison with not irradiated *A. albopictus* males (0.46 ± 0.08) marked with higher quantity of fluorescent dust and released in similar environments in previous MRR trials (Bellini et al., 2010). A similar daily survival rate (0.79 ± 0.24) was instead observed in comparison with fertile males marked without the use of fluorescent dust (aposymbiotic strain) (Bellini et al., 2010).

Using the Lincoln index, the overall mean size (±SD) of the population estimated in Caselle (89,632 ± 44,795) and in Guisa Pepoli (44,795 ± 21,622) were different (F_1,14_ = 15.15; *p* = 0.0016) with an higher mean number of male per ha (F_1,14_ = 5.09; *p* = 0.041) observed in Caselle (7,091 ± 4,054) in comparison with Guisa Pepoli (3,024 ± 3,089). The mean value (SD) of the daily population size and density estimations for each group of mosquitoes marked with different colors are reported in Table 2.

The overall mean (±SD) residual fertility of *A. albopictus* males irradiated at 35 Gy at the pupal stage and measured on caged individuals in laboratory conditions (Balestrino et al., 2010) was equal to 1.00 ± 0.72% (Supplementary Materials S1–S4). According to the model developed by Aronna and Dumont (2020), it is necessary to limit the residual fertility of the released males according to the population basic offspring number and anyway below 2% to achieve an effective control of *A. albopictus* populations in a tropical area using the SIT as part of an integrated pest management (IPM) approach. Considering a population abundance similar to that described for the same tropical areas (Erguler et al., 2017), the use of radiation doses between 30 and 40 Gy is confirmed to be the most effective dose to effectively induce sterility into the natural population with an acceptable residual fertility lower than 2% (Balestrino et al., 2010; Bellini et al., 2013a; Bellini et al., 2021). At these doses the mating competitiveness of males and the effectiveness of the SIT technique for the suppression of *A. albopictus* populations has already been validated in the laboratory (Balestrino et al., 2017; Culbert et al., 2018) and in field temperate areas (Bellini et al., 2013a; Bellini et al., 2021). The overall mean (±SD) natural fertility calculated in the untreated control area of Bolognina during the MRR study period was 91.6 ± 3.8, while in the release sites the observed fertility was equal to 73.1 (±4.69) and 86.8 (±8.50), respectively, in Guisa Pepoli and Caselle (Table 1). The fertility rate of the eggs collected in Caselle and in Guisa Pepoli during the MRR trials were all lower than the fertility eggs registered in the control area of Bolognina in the same periods. The sterile male release rates achieved in these trials were equal to 912–458 males/ha in Caselle and 868–2,486 in Guisa Pepoli, respectively, in MRR1 and MRR2 (Table 1, NA_M__R/ha). However, the fertility rate difference observed in the eggs collected in the field was significant only in Guisa Pepoli during the MRR2 (F_1,12_ = 24.4, *p* = 0.0003). The competitiveness index was calculated in Caselle and Guisa Pepoli during the first and the second MRR trials (range 0.25–0.49; Table 1) even if the only significant value for this parameter was collected in Guisa Pepoli during MRR2 (0.43, Table 1).

## Discussion

The mass rearing methods applied at the CAA facilities in 2018 allowed a male recovery rate of about 20% of the reared males, with a mean female contamination of 1.65%. These rearing parameters are currently one of the most limiting factors affecting the development of a cost-effective large-scale implementation of SIT against *A. albopictus*. The females accidentally irradiated and released with males become permanently sterilized (Balestrino et al., 2010) but still maintain their biting activity with a short-term risk of increased arboviral disease transmission. Even if the accidental release of females could not sensibly increase the epidemiological risk of disease transmission and do not seriously affect the SIT effectiveness (Dumont and Yatat-Djeumen, 2022), their presence could affect the political and ethical acceptability of this technique, especially in areas where mosquito-borne diseases are endemic, hence the recommendation to keep it below 1% (WHO/IAEA, 2020). A more effective and consistent sex sorting system is therefore strongly needed to increase the male productivity and the sex separation accuracy, as pointed out recently (Lutrat et al., 2019). In a recent successful trial in China, the authors reported a female contamination of 0.3% for a male recovery of 70% which offers new perspective for future SIT trials (Zheng et al., 2019).

As previously showed (Carrieri et al., 2011; Bellini et al., 2013b), the ovitrap monitoring system as employed in the three study localities can provide a good estimation of the *A. albopictus* wild population density. The authors of this study reported a highly positive correlation between the number of pupae per hectare (PHI) and the weekly mean number of eggs per ovitrap collected in similar field environment in northern Italy. The distribution and availability of competitive artificial containers could modify the female oviposition behavior in ovitraps, thus influencing the relationship between the number of females and the number of eggs in the ovitraps. However, according to the findings described by Carrieri et al. (2011), in *A. albopictus*, the ovipositing females did not seem to be influenced by the egg density in the available breeding sites. This aspect together with the uniform distribution of the most productive larval habitats (catch basins) in Italy allowed the effective use of egg density data from ovitraps to infer the mean number of adults per unit area. A significant positive relationship between ovitrap data and data from HLC was also reported in field trials carried out in the metropolitan area of Rome, Italy (Manica et al., 2017). The authors of this study confirmed the possibility to successfully predict the mean number of adult biting females in HLC based on the mean weekly number of eggs collected in the field. Our results, based on a limited data set, are in line with these results and support the conclusion that the relationship between the number of eggs and the wild adult population can be effectively and significantly established in Italy thanks to the capillary distribution of the available breeding sites, while a more generalized relationship between eggs density in ovitraps and wild adult population cannot be inferred for different environment without specific field investigations. The BG-sentinel traps activated for 24 h once a week as employed in our studies produced data not sufficiently accurate to effectively estimate the adult population dynamics in the study areas. However, daily sampling comparison between BG-sentinel trap baited with BG-Lure and CO2 and HLC method demonstrated similar trapping efficiency and provides similar estimations of the main entomological parameters during MRR trials with *Aedes albopictus* E. Velo, personal communication.

The mean recapture rates obtained in our trials (0.27–1.7%), despite the large variability observed between localities and sessions, may be considered within the range usually found in mark-release-recapture studies (Service 1993; Caputo et al., 2021) and is not different from values observed in *A. albopictus* fertile males marked with fluorescent dust in the same environment with similar recapture density (Bellini et al., 2010).

The recapture rate strongly depends on the dispersal capacity of the target species, on the recapture effort (density of recapture stations) and on the efficacy of recapture method employed (Service 1993).

The radio-sterilized *A. albopictus* males showed an overall MDT and survival rate not different from values registered for this species in different MRR studies with either sterile or fertile individuals (Bellini et al., 2010; Gouagna et al., 2015; Iyaloo et al., 2019). It is interesting to note that the overall MDT value obtained in this study using radio-sterilized *A. albopictus* males marked with fluorescent powders (MDT = 149.6 ± 48.9 m) is not different (*p* = 0.51) from the MDT obtained with not irradiated *A. albopictus* males (MDT = 124.4 ± 21.2 m) similarly marked with fluorescent dust and released in similar environments (Bellini et al., 2010).

While we cannot exclude that fluorescent dust-based marking procedures may sensibly increase mortality and decrease mobility of marked mosquito (Dickens and Brant, 2014), the similar field performances obtained between sterile and fertile *A. albopictus* males released in similar environmental condition indicate the negligible effects of the irradiation dose (35 Gy) on the quality of the sterile males released.

In this study we confirmed the positive linear relationship between sterile adult male survival rate (s) and relative humidity (RH), as previously reported (Bellini et al., 2010; Bellini et al., 2021). Adult dehydration caused by high temperatures and low RH is likely to be an important factor affecting survival (Costa et al., 2010) and could influence the population size of these insects in the environment (Alto and Juliano 2001).

However, the observed survival and dispersion seems to be influenced more by environmental factors such as barriers, shading and vegetation rather than weather parameters. Among the environmental factors, the vegetation coverage can strongly influence the abundance of *A. albopictus* and can positively affect their dispersal capacity and distribution (Ayllón et al., 2018; Iyaloo et al., 2019). The positive correlation we observed between the NDVI index and the sterile male density confirm the important role of the vegetation coverage in the dispersal of the released *A. albopictus* males. The sterile males need to disperse from the release sites to reach natural courtship and mating arenas where wild males and females are already present. Soon after the release, the sterile males are still more concentrated and active in the proximity of the release sites while not yet dispersed in more distant areas. Consistent with previous studies (Bellini et al., 2010), in the first day following the release males have their maximal dispersion while in the following days the males move less and with a reduced directionality.

The S/W ratio measured showed a large variability and its variance is higher than the mean value suggesting a cluster distribution of the released sterile males, at least until the second or third day after release. The S/W male ratio measured in these MRR field trials, was also used to determine the mean (±SD) sterile male competitiveness in Guisa Pepoli and Caselle which confirmed the effective mating capacity of the released radio-sterilized males in the field (Bellini et al., 2021). Mass production, manipulation, irradiation and transportation procedures can affect the performance of the sterile adult males in the field. The competitiveness indices calculated during these trials indicate an effective mating behavior of the released sterile males in the target field areas even if they should be interpreted with caution since they were reported for a single week of release and differences in hatch rates between the control and release areas were not all significant. However, in operational SIT release campaigns performed in the same environments a release rate of 896–1,590 males/ha per week was effective to suppress wild populations (Bellini et al., 2013a) with effective field competitiveness (Bellini et al., 2021). The sterile male field competitiveness is directly affected by the spatial distribution of the sterile and wild males in the field and a release method which allows a more homogeneous distribution of the sterile males in the field could probably assist the SIT programs effectiveness. The possibility to release sterile adult mosquitoes males by a drone was already demonstrated for Aedes mosquitos in the field with substantial reduction of the operational release costs (Bouyer et al., 2020). The aerial release systems would also allow males to be released in a closely controlled manner into specific geographic locations difficult to access and according with the local population density.

## Data Availability

The original contributions presented in the study are included in the article/Supplementary Material; further inquiries can be directed to the corresponding author.
